# Biomolecular imaging of colorectal tumor lesions using a FITC-labeled scFv-Cκ fragment antibody

**DOI:** 10.1038/s41598-021-96281-z

**Published:** 2021-08-25

**Authors:** Hyung Il Kim, Jinhyeon Kim, Hyori Kim, Hyeri Lee, Yong Sik Yoon, Sung Wook Hwang, Sang Hyoung Park, Dong-Hoon Yang, Byong Duk Ye, Jeong-Sik Byeon, Suk-Kyun Yang, Sun Young Kim, Seung-Jae Myung

**Affiliations:** 1 Department of Medical Science, Asan Medical Institute of Convergence Science and Technology, Asan Medical Center, University of Ulsan College of Medicine, Seoul, Republic of Korea; 2 Edisbiotech, Songpa-gu, Seoul, Republic of Korea; 3 Convergence Medicine Research Center, Asan Medical Center, Seoul, Republic of Korea; 4 Department of Colon and Rectal Surgery, Asan Medical Center, University of Ulsan College of Medicine, Seoul, Republic of Korea; 5 Department of Gastroenterology, Asan Medical Center, University of Ulsan College of Medicine, Seoul, Republic of Korea; 6 Asan Institute for Life Sciences, Asan Medical Center, University of Ulsan College of Medicine, 88, Olympic-ro 43-gil, Songpa-gu, Seoul, 05505 Republic of Korea; 7 Digestive Diseases Research Center, University of Ulsan College of Medicine, Seoul, Republic of Korea

**Keywords:** Biological techniques, Cancer, Molecular biology, Gastroenterology, Oncology

## Abstract

For the sensitive diagnosis of colorectal cancer lesions, advanced molecular imaging techniques using cancer-specific targets have emerged. However, issues regarding the clearance of unbound probes and immunogenicity remain unresolved. To overcome these limitations, we developed a small-sized scFv antibody fragment conjugated with FITC for the real-time detection of colorectal cancer by in vivo molecular endoscopy imaging. A small-sized scFv fragment can target colon cancer secreted protein-2 (CCSP-2), highly expressed in colorectal adenocarcinoma tissues; moreover, its full-length IgG probe has been used for molecular imaging previously. To assess the efficacy of anti-CCSP-2 scFv-FITC, surgical specimens were obtained from 21 patients with colorectal cancer for ex vivo molecular fluorescence analysis, histology, and immunohistochemistry. Orthotopic mice were administered with anti-CCSP-2 scFv-FITC topically and intravenously, and distinct tumor lesions were observed by real-time fluorescence colonoscopy. The fluorescence imaging of human colon cancer specimens allowed the differentiation of malignant tissues from non-malignant tissues (*p* < 0.05), and the CCSP-2 expression level was found to be correlated with the fluorescence intensity. Here, we demonstrated the feasibility and safety of anti-CCSP-2 scFv-FITC for molecular imaging as well as its potential in real-time fluorescence colonoscopy for the differential diagnosis of tumor lesions.

## Introduction

Colorectal cancer is known as one of the most common cancers and causes of cancer-related death worldwide, and more than 1,000,000 cases are detected annually^1^. Advances in early diagnosis and treatment options have led to a decline in colorectal cancer mortality despite increasing incidence rates^2^. A number of studies have reported improvements in the quality of gastrointestinal endoscopy^3–6^. However, recent studies disputed the success and effect of surveillance colonoscopy in some patients such as intermediate-risk patients^7–9^ and reported the inaccurate delineation of non-polypoid lesions^10,11^. Conventional white-light colonoscopy has high sensitivity; however, it tends to miss small, flat, or depressed lesions that are potentially invasive, resulting in progression to advanced tumors^12–14^. Additionally, the early detection of cancer in patients with long-term ulcerative colitis or Crohn’s disease by conventional colonoscopy is challenging^15,16^. Colitis-related colorectal cancer lesions are usually multifocal and flat and difficult to distinguish from chronic colitis-associated background inflammation^17,18^. Therefore, more sensitive imaging-based tumor lesion detection techniques are needed.

In the past years, for the sensitive diagnosis of colorectal cancer lesions, advanced molecular imaging techniques such as autofluorescence imaging, near-infrared imaging, and confocal endomicroscopy/pCLE have emerged^19–21^. As molecular imaging is based on externally derived probes labeled with a fluorescent dye or other markers, various probes for molecular imaging in the gastrointestinal tract have been studied. Boodgerd et al. evaluated the first clinical use of a fluorophore-labeled antibody targeting carcinoembryonic antigen (CEA) for the detection of colorectal cancer during surgery^22^. Burggraaf et al. used a peptide that can bind to the human tyrosine kinase c-Met to identify colorectal cancer through fluorescence colonoscopy in 15 patients with a high risk of colorectal neoplasia^23^. These studies demonstrated the potential of the clinical application of fluorescently labeled probes for cancer diagnosis; however, a long half-life after injection is an issue associated with the intravenous (i.v.) administration of probes.

Previously, we have reported that a fluorescent dye-conjugated antibody targeting colon cancer secreted protein-2 (CCSP-2), a protein highly expressed in colorectal adenoma and adenocarcinoma tissues, may be used to distinguish cancer lesions and normal tissues with fluorescent signals that could be detected by ex vivo molecular imaging^24^.

We generated a single-chain variable fragment (scFv) specific to CCSP-2 for detecting human colorectal cancer lesions. As scFv fragments, which can recognize the same antigens as IgG antibodies, are designed for rapid target binding in molecular imaging^25^, the injection of scFv fragments allows penetration into tissue complexes and the rapid binding and release of antigens^26^.

In the present study, we describe the development and characterization of FITC-conjugated anti-CCSP-2 scFv, a novel fluorescent probe for detecting colorectal cancer lesions by fluorescence colonoscopy. We validated the use of scFv-FITC to target colorectal cancer lesions in an orthotopic murine model by fluorescence colonoscopy with high sensitivity within 30 min. In addition, we assessed its ability in detecting colorectal tumors in patients with primary colorectal cancer by ex vivo molecular imaging.

## Results

### Generation, purification, and characterization of anti-CCSP-2 scFv antibody fragment

**Figure 1:**
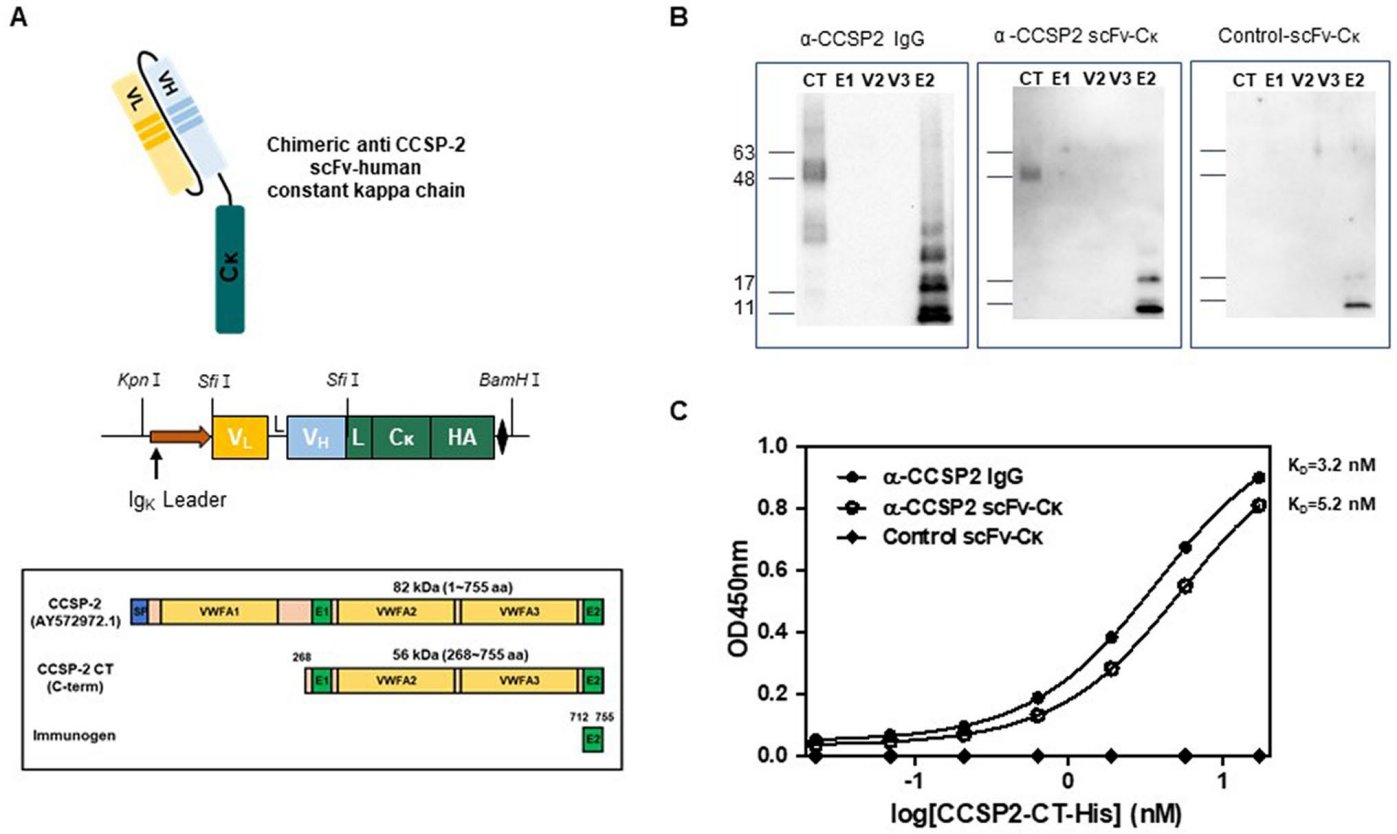
Generation and characterization of anti-CCSP-2 scFv antibody. (**A**) Schematic illustration of human kappa chain labeled with scFv and CCSP-2 domains. (**B**) Analysis of the binding affinity between CCSP-2 domains and anti-CCSP-2 antibodies by western blotting. (**C**) Equilibrium binding assay of anti-CCSP-2 IgG and scFv-C kappa (Cκ) with histidine-labeled CCSP-2 CT (CCSP-2 CT-His).

We constructed an immunogen from CCSP-2 (E2; EGF-like domain 2, amino acid 712–755) including the binding region of anti-CCSP-2 IgG for phage display using a chicken library (Fig. 1A, Supplementary Fig. S1). Purified scFv clones were subjected to western blotting with several domains from CCSP-2 including the C-terminal domain (CCSP-2 CT, 56 kDa), which binds to anti-CCSP-2 IgG. We selected scFv with a high binding reactivity with the CCSP-2 CT antigen compared with anti-CCSP-2 IgG, and CCSP-2 E2 domain (5 kDa) was used as a positive control (Fig. 1B). To further confirm the affinity, anti-CCSP-2 scFv, anti-CCSP-2 IgG, and control scFv were subjected to enzyme immunoassay. The selected anti-CCSP-2 scFv and anti-CCSP-2 IgG clones showed high affinity with K_D_ values of 5.2 nM and 3.2 nM, respectively (Fig. 1C, Table S1).

### Binding specificity of FITC-labeled scFv in vitro and in vivo

**Figure 2:**
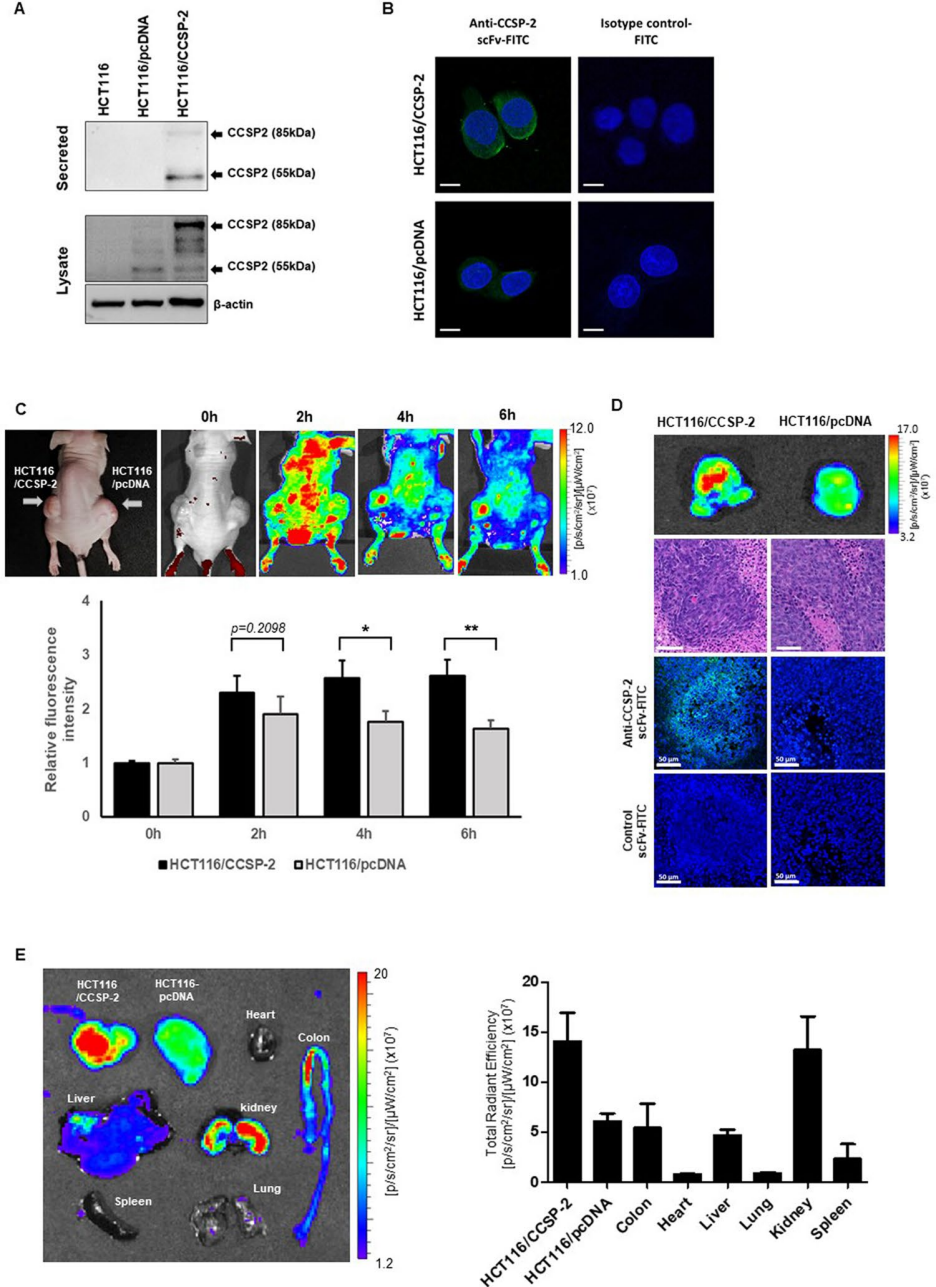
Molecular imaging of CCSP-2 probe binding in vitro and in vivo. (**A**) Western blot analysis of CCSP-2 in HCT116 cells transfected with a CCSP-2-encoding plasmid. (**B**) In vitro confocal images of CCSP-2-overexpressing HCT116 cells and control vector-transfected HCT116 cells treated with FITC-conjugated anti-CCSP-2 scFv. FITC-conjugated kappa isotype control antibody was used as the negative control (magnification: ×630, scale bar: 10 μm). (**C**) In vivo molecular images of CCSP-2-overexpressing xenograft tumor-bearing mice after the tail vein injection of anti-CCSP-2 scFv-FITC. Data shown are the mean ± SD of at least three independent experiments (*p < 0.05, **p < 0.01, n = 6). (**D**) Isolated solid tumors acquired from sacrificed mice to examine the fluorescence intensity ex vivo (top) and representative histological and immunofluorescence staining of xenograft tumors (original magnification: ×200, scale bar: 50 μm). (**E**) Ex vivo biodistribution analysis by fluorescence intensity. Organs were removed from anti-CCSP-2 scFv-FITC-injected mice after 8 h (n = 6).

The selected scFv had the optimal yields of soluble FITC-labeled scFv antibody (anti-CCSP-2 scFv-FITC) and was further investigated for molecular imaging analysis. We used previously established CCSP-2-overexpressing HCT116 cells (HCT116/CCSP-2)^24^. The CCSP-2 expression of cultured supernatants and cell lysates was detected at 55 kDa and 85 kDa (associated with secreted and full-length CCSP-2, respectively) by western blotting (Fig. 2A). To validate the binding specificity and internalization of scFv antibodies, the localization of scFv antibodies in CCSP-2-overexpressing HCT116 cells was examined by confocal microscopy. As shown in Fig. 2B, anti-CCSP-2 scFv-FITC was accumulated in HCT116/CCSP-2 cells; however, there was no signal in HCT116/pcDNA cells (control). In addition, we performed immunocytochemistry without a permeabilization procedure, and we observed the accumulation of anti-CCSP-2 scFv-FITC on the cell surface and partly in the cytoplasm in the CCSP-2-overexpressing cell line. Next, to evaluate the effectiveness and specificity of anti-CCSP-2 scFv-FITC in targeting tumors in vivo, molecular imaging was performed using a subcutaneous xenograft murine model. After 4 and 6 h from the i.v. injection of FITC-labeled anti-CCSP-2 scFv, HCT116/CCSP-2-derived tumors showed significantly greater fluorescence intensity compared with the intensity of HCT116/pcDNA-derived tumors (Fig. 2C). However, when the FITC control dye was injected into the mice, there was no distinct accumulation of fluorescence signals in HCT116/CCSP-2- and HCT116/pcDNA-derived tumors (Supplementary Fig. S2). The tumors were removed at 8 h post-injection for further fluorescence imaging, and a high fluorescent intensity was clearly observed in HCT116/CCSP-2-derived tumors compared with HCT116/pcDNA-derived tumors. The results of the anti-CCSP-2 scFv-FITC staining and H&E staining of the tumor tissue sections were consistent with in vivo observations (Fig. 2D).

A toxicology analysis of a single dose of CCSP-2 scFv-FITC in mice was performed, and there were no probe-related acute effects on clinical signs, hematology, or clinical chemistry (Supplementary Table S2). The total fluorescence intensity was increased from 0 to 2 h and was gradually decreased from 2 h after the injection of anti-CCSP-2 scFv-FITC in the murine model. The half-life of the probe was 3.6 h (Supplementary Fig. S3). The biodistribution of anti-CCSP-2 scFv-FITC after 8 h from i.v. injection is shown in Fig. 2E. Increased fluorescence signals in HCT116/CCSP-2-derived tumors were confirmed; however, the signals in other organs except the kidney were decreased.

### Fluorescence colonoscopy of colorectal cancer lesions in orthotopic mice

**Figure 3:**
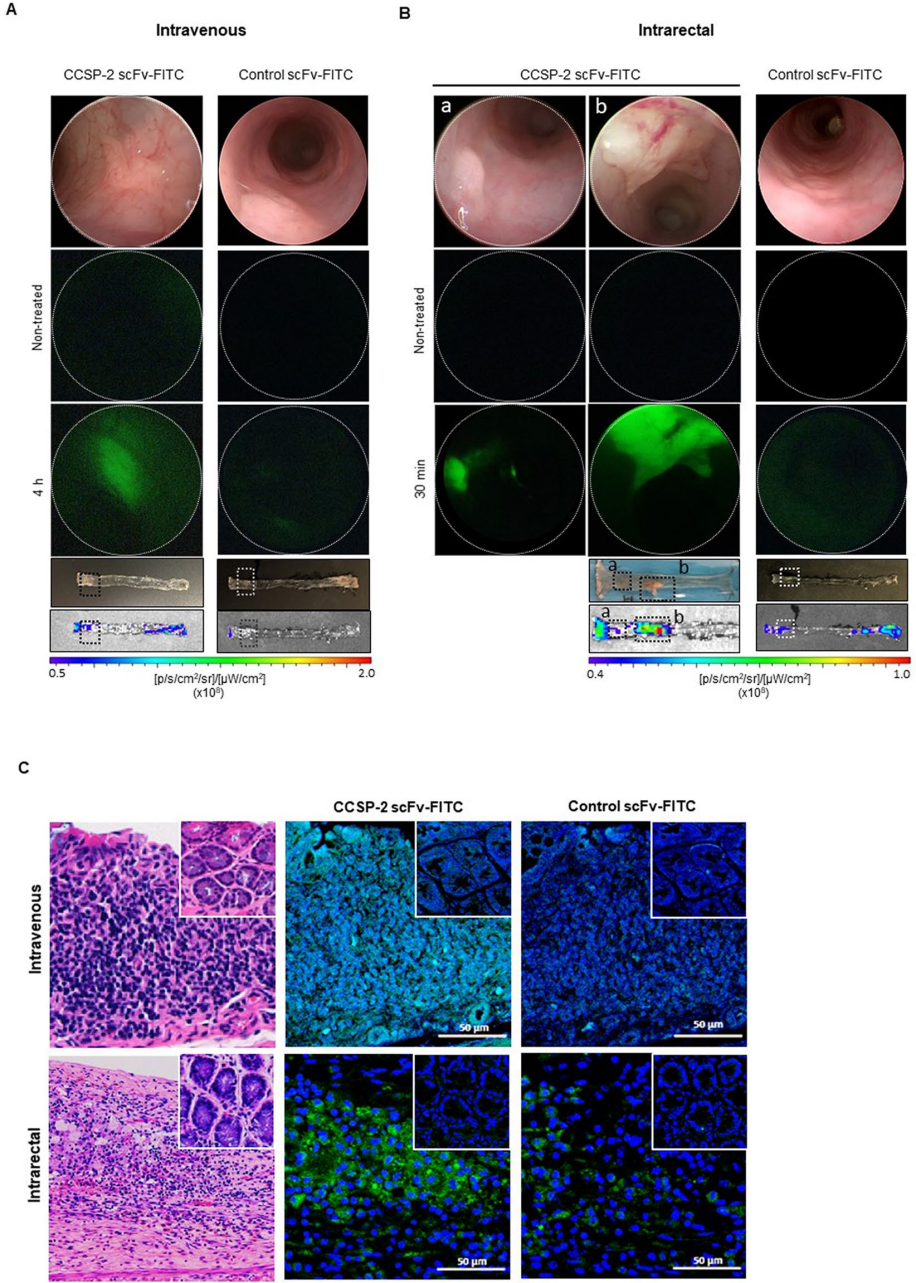
In vivo CCSP-2-targeted fluorescence colonoscopy in an orthotopic murine model. (**A**) Fluorescence images showing orthotopic cancer growth in a region with increased intensity with minimal background fluorescence in an anti-CCSP-2 scFv-FITC or control scFv-FITC-treated lesion compared with a non-treated lesion. The images were obtained 4 h after the i.v. injection of anti-CCSP-2 scFv-FITC or control scFv-FITC. Real-time white-light image of the tumor lesion was also acquired. (**B**) Fluorescence images showing distinct orthotopic cancer growth in a region with increased intensity in an anti-CCSP-2 scFv-FITC or control scFv-FITC-treated lesion compared with a non-treated lesion. The images were obtained 30 min after intrarectal administration of anti-CCSP-2 scFv-FITC or control scFv-FITC. Real-time white-light image of the tumor lesion was also acquired. (**C**) Histological analysis of tumor tissues from orthotopic xenograft mice injected with CCSP-2-overexpressing HCT116 cells. The inner square shows the adjacent normal tissues.

We established an orthotopic murine model with tumor growth in the colon mucosa. After the injection of CCSP-2-overexpressing cells (HCT116/CCSP-2), tumor growth was observed by white-light colonoscopy for 4 weeks, which was confirmed to be a carcinoma by histological analysis (Supplementary Fig. S4, Supplementary Video S1). To determine whether anti-CCSP-2 scFv-FITC was effective in targeting tumor lesions in vivo, orthotopic tumor-bearing mice were evaluated by fluorescence colonoscopy. Mice with confirmed tumor growth were injected with anti-CCSP-2 scFv-FITC (1.5 µg/g body weight) via the tail vein. After 4 h, we could observe the accumulation of scFv-FITC signals in tumor lesions with minimal autofluorescence background (Fig. 3A). We also administered scFv-FITC intrarectally to mimic the spraying of probes; at 10 ~ 30 min after administration, distinct tumor lesions were observed by real-time fluorescence colonoscopy (Fig. 3B, Supplementary Video S2). When the control scFv-FITC was injected into the mice, there was no distinct accumulation of fluorescence signals in tumors and adjacent normal. The fluorescence intensity value of the region of interest (ROI) was quantified; the average fluorescence intensity values of tumors from intravenously and intrarectally treated mice were 1.25 × 10^8^ [p/s/cm^2^/sr]/[μW/cm^2^] and 4.24 × 10^8^ [p/s/cm^2^/sr]/[μW/cm^2^], respectively (Fig. 3A,B, bottom panel). Immunofluorescence staining of CCSP-2 in tissue paraffin sections confirmed tumor epithelial cell-specific binding and the absence of signals in adjacent normal cells (Fig. 3C).

### Molecular imaging of human tissues from colorectal cancer patients

**Figure 4:**
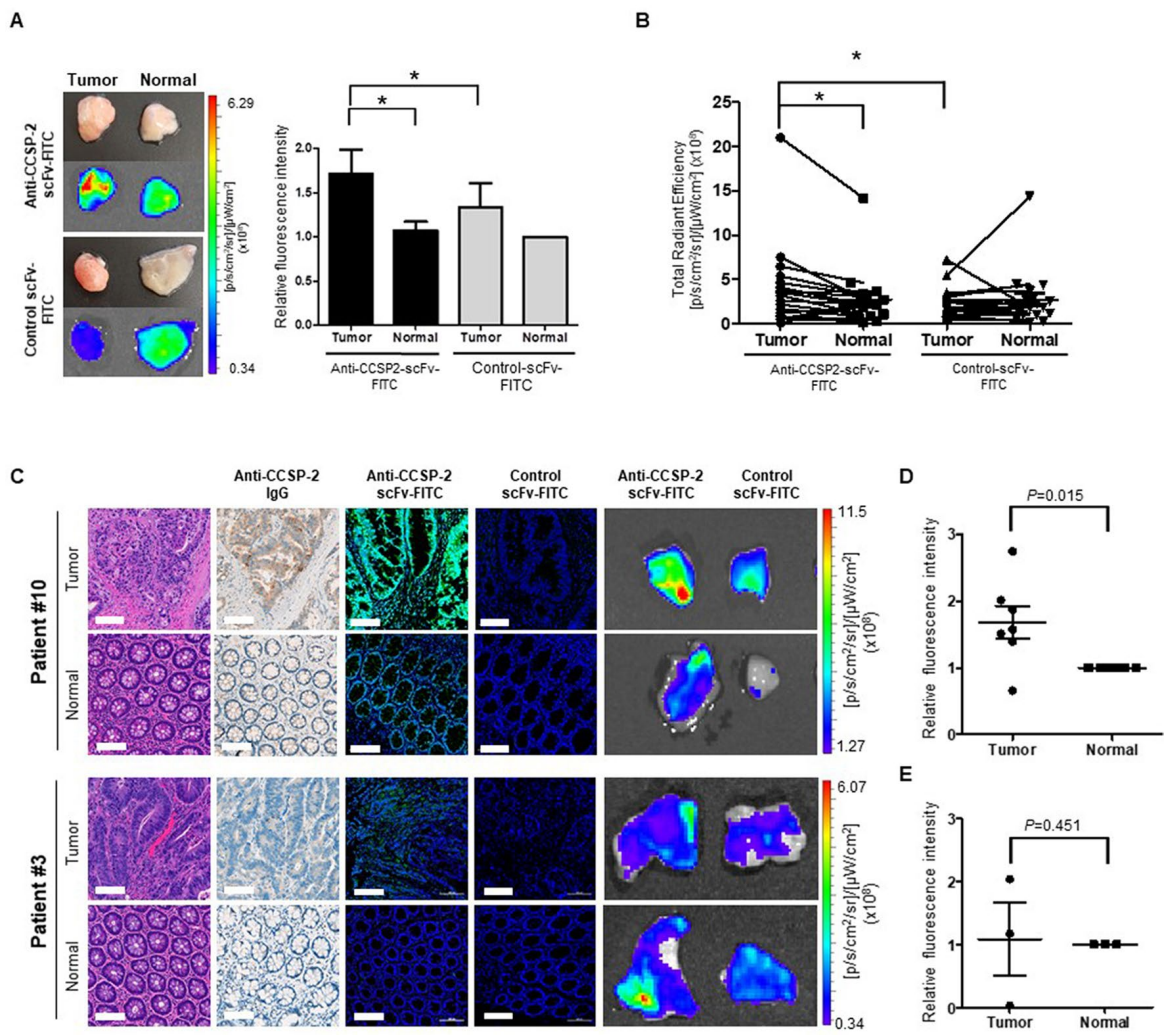
Ex vivo molecular imaging of tumor tissues from colorectal cancer patients. (**A**) Representative ex vivo fluorescence imaging and relative fluorescence intensity graph of colorectal cancer patient specimens treated with FITC-conjugated anti-CCSP-2 scFv and control scFv (*p < 0.05). (**B**) Total fluorescence intensity of each patient specimen treated with FITC-conjugated anti-CCSP-2 scFv and control scFv (*p < 0.05). (**C**) Representative H&E, IHC, and immunofluorescence imaging of colorectal cancer patient tissues with strong (upper panel) and weak (bottom panel) expression of CCSP-2. (**D**) Relative fluorescence intensity of patient tissues with strong expression of CCSP-2 (n = 7). (**E**) Relative fluorescence intensity of patient tissues with weak expression of CCSP-2 (n = 3). Data shown are the mean ± standard error of the mean (SEM).

We analyzed the efficacy of the anti-CCSP-2 scFv-FITC probe using a total of 42 samples from 21 patients with colorectal cancer (Table 1) by ex vivo molecular imaging. The fluorescence intensity of the tumor/normal tissues including frozen and surgical samples was evaluated after incubation with anti-CCSP-2 scFv-FITC or control scFv-FITC. In comparison with adjacent normal tissues, colorectal tumor tissues from patients incubated with anti-CCSP-2 scFv-FITC showed a significantly higher fluorescence intensity (71%, *p* = 0.0342) (Fig. 4A). The total fluorescence intensity was analyzed to evaluate the feasibility for in vivo colonoscopy. The total fluorescence intensity of tumor tissues with anti-CCSP-2 scFv-FITC was increased compared with that of normal tissues (15/21 patients, 71%, *p* = 0.0207), and no significant differences were detected between tumor and normal tissues with control scFv-FITC (Fig. 4B). The total fluorescence intensity of tumor tissues with anti-CCSP-2 scFv-FITC was higher than that of tumor tissues with control scFv-FITC from 15 patients (71%, *p* = 0.0365, Fig. 4B). The incubation time for frozen tissues was shorter (15 min) than that for surgical fresh tissues (30 min); a longer incubation with antibodies may degrade the frozen tissues, resulting in an overall higher reactivity with the probes (Supplementary Fig. S5). Immunofluorescence staining with anti-CCSP-2 scFv-FITC detected the expression of CCSP-2 in patient colorectal cancer tissues (consistent with IHC data); however, there was no significant fluorescence in both tumor and normal tissues with control scFv-FITC (Fig. 4C). The fluorescence signal was mainly localized in the cytoplasm of the tumor epithelial cells, which was in agreement with our previous observations and immunohistochemistry of the same tissue sections with anti-CCSP-2 IgG. The fluorescence intensity was analyzed based on the CCSP-2 expression level of fresh tumor samples to validate the specificity of the probe. Although the fluorescence intensity was higher in tumor samples with strong and weak CCSP-2 expression than in paired normal samples, the difference was significantly greater in samples with strong expression (*p* = 0.015) than in samples with weak-negative expression (*p* = 0.451) of CCSP-2 (Fig. 4D,E).

**Table 1:** Clinical information of colorectal cancer patients.

Characteristic	No. of patients (n = 21)	%
**Gender**		
Male	11	52
Female	10	48
**Age**		
≤ 55	12	57
> 55	9	43
**Histological grade**		
Adenoma	5	24
Well differentiation	2	9
Moderate differentiation	14	67
Poor differentiation	0	0
**Metastasis**		
ND	15	71
Lymph node	6	29

## Discussion

In this study, we demonstrated the feasibility of visualizing colorectal lesions by real-time fluorescence colonoscopy using a novel scFv fragment targeting CCSP-2 in a pre-clinical model of human colorectal cancer. The detection ability of anti-CCSP-2 scFv-FITC for CCSP-2-overexpressing colorectal cancer lesions was evaluated through both topical and i.v. administration in an orthotopic murine model, and we investigated its efficacy with human samples. Anti-CCSP-2 scFv is a single-chain variable fragment that can target CCSP-2. CCSP-2 was found to be overexpressed in nearly 90% of colon adenomas and cancers in our previous study; thus, it is a notable target for molecular fluorescent probes.

A number of studies have attempted to detect colorectal cancer lesions using fluorescently labeled antibodies against surface markers such as epidermal growth factor receptor (EGFR), CEA, or vascular endothelial growth factor (VEGF)^27–29^. In comparison with nonspecific dyes, these antibodies have demonstrated highly specific binding affinity with stronger signals; however, issues related to the clearance of unbound probes and immunogenicity remain unresolved. To overcome these limitations, the use of small molecular probes has been suggested for enhanced binding kinetics, efficient penetration into the altered mucosa, favorable tissue distribution patterns, reduced immunogenicity, and low toxicity^23,30–32^. In the present study, we constructed a small molecular antibody fragment targeting CCSP-2, which showed high affinity and specificity comparable to those of full-length IgG antibody in vitro and in vivo. The anti-CCSP-2 scFv-FITC probe was detected in the cytoplasm of fixed human colorectal cancer cells without a permeabilization procedure, and the optimal incubation time of the probe in ex vivo imaging with human specimens was shorter (30 min) than that of the full-length IgG antibody probe from our previous study (1 h). Intravenously injected anti-CCSP-2 scFv-FITC was highly accumulated in the CCSP-2-overexpressing tumors of mice. In addition, single-dose toxicity and total fluorescence half-life analysis showed the safety of the probe in these mice. These results suggest that anti-CCSP-2 scFv-FITC may be used for the visualization of colorectal cancer lesions in clinical settings.

Pre-clinical cancer models are essential for the clinical translation of molecular imaging probes, and previous studies have shown that orthotopic cancer models are necessary for ectopic evidence^33^. In this study, we successfully established an orthotopic murine colorectal cancer model via colonic submucosal injection to provide a more accurate pre-clinical model. We introduced the probe to this orthotopic murine colorectal cancer model both topically and intravenously for molecular fluorescence colonoscopy. As the clear detection of lesions is one of the major requirements of a molecular probe to avoid additional risks and costs related to the removal of non-neoplastic lesions^34^, we aimed to accurately identify tumor lesions by fluorescence colonoscopy. In conventional confocal laser endomicroscopy (CLE) imaging of colorectal cancer tissues, avoiding mucosal penetration with adequate staining time is important for an accurate analysis; thus, i.v. application rather than topical application has been recommended^35,36^. For wide-field lesion detection, topical application is advantageous for reducing immunogenicity and rapid clearance; however, this is not practical in the colon, and there are limitations associated with mucosal binding and penetration^23,37,38^. Therefore, in the current study, we developed a small molecular antibody fragment with high affinity, and we observed distinct tumor lesions in the murine model with topically administered anti-CCSP-2 scFv-FITC by fluorescence molecular imaging within 30 min. Although the i.v. application of anti-CCSP-2 scFv-FITC showed a reliable fluorescence signal, it resulted in a relatively longer incubation time and slightly lower fluorescence intensity. These results demonstrated the efficacy of the topical administration of anti-CCSP-2 scFv-FITC for detecting macroscopic lesions through real-time fluorescence colonoscopy.

To investigate the efficacy of the topical administration of anti-CCSP-2 scFv-FITC in humans, human tissue specimens were incubated with the probe. We identified 21 adenocarcinoma lesions from primary colon cancer patients, and most of these (71.4%, 15/21) showed increased fluorescence compared with the fluorescence of non-neoplastic tissues. Furthermore, examination of human tissues or orthotopic models of colorectal carcinoma revealed that the effects of luminal factors should be minimized, which could interfere with antibody binding after topical administration. The results of immunofluorescence staining with anti-CCSP-2 scFv-FITC was well correlated with molecular imaging results.

In our previous study, fluorescently conjugated full-length antibodies against CCSP-2 were used to evaluate targeting efficacy and specificity^24^. The findings suggest that CCSP-2 could serve as an important candidate biomarker for tumor imaging, as demonstrated by IHC data in the present study. Additionally, compared with full-length antibodies, anti-CCSP-2 scFv-FITC has some advantages due to its small molecular size, ease of synthesis, and low immunogenicity. Single-chain variable fragments can be developed for potential therapeutic applications. Sokolowska-Wedzina et al.^39^ reported the advantages of the Fc-fusion format of scFv fragments, which include higher tumor uptake^40^. Moreover, small molecular scFv antibody fragments have shown Fc-mediated effector functions^41^ and convenient drug conjugation possibilities.

In this study, we demonstrated that anti-CCSP-2 scFv-FITC could be an ideal probe for tumor imaging; however, there are some challenges in clinical use. A small sample number without adenoma cases could limit the generalizability of the results. Its feasibility in detecting early-phase carcinoma in a larger cohort needs to be evaluated. Although the general toxicity and total fluorescence half-life analyses in this study may indicate the safety of the probe, a more extensive assessment would be needed for the clinical use of anti-CCSP-2 scFv-FITC.

In conclusion, we demonstrated the feasibility and safety of anti-CCSP-2 scFv-FITC for molecular imaging as well as its potential in real-time fluorescence colonoscopy for the differential diagnosis of tumor lesions.

## Material and methods

### Patient consent and study inclusion

The study was approved by the Institutional Review Board of Asan Medical Center (IRB; protocol No. 2017-0837) and performed according to the ethical principles of the Declaration of Helsinki. All patients gave written informed consent before being included in the study.

### Animal study ethics

All animal studies were approved by Animal Care and Use Committee (IACUC; Approval No. 2018-12-151) of Asan Medical Center. All animal care and experimental procedures were carried out in accordance with the relevant guidelines and regulations of the IACUC of Asan Medical Center and ARRIVE guidelines.

### Generation of anti-CCSP-2 scFv antibody

Three White Leghorn chickens were immunized and boosted three times with 20 μg of CCSP-2-His protein. The spleen, bursa of Fabricius, and bone marrow were harvested for total RNA isolation using TRI Reagent (Invitrogen, CA, USA). Complementary DNA was synthesized using the Superscript IV First-Strand Synthesis system (Invitrogen, CA, USA) to generate scFv-displaying phage libraries as described previously (C.F. Barbas, Phage Display: A Laboratory Manual, Cold Spring Harbor Laboratory Press, Cold Spring Harbor, NY, 2001). The libraries were subjected to five rounds of bio-panning with CCSP-2-conjugated magnetic beads. Individual phage clones were selected randomly from the output titration plate of the last round, and scFv-displaying phages were subjected to enzyme-linked immunosorbent assay (ELISA) as described previously^42^.

### Expression and purification of anti-CCSP-2 scFv-Cκ fusion protein

The gene encoding anti-CCSP-2 scFv was subcloned into modified pCEP4 mammalian expression vectors to express human constant kappa region (Cκ) fusion proteins as described previously^42^. The C-terminal cysteine residue of Cκ was excluded to abolish the dimerization of scFv-Cκ fusion proteins through disulfide bond formation. The construct was transfected into HEK293F cells, and scFv-Cκ fusion proteins were purified using KappaSelect Resin (GE Healthcare) as described previously^43^.

### Anti-CCSP-2 scFv-FITC conjugation

FITC conjugation was performed by BioActs (Incheon, Korea). Antibodies were dissolved in 0.1 M carbonate-bicarbonate buffer (pH 9.5), and FITC-NHS ester in anhydrous dimethylformamide (DMF) was added for NHS-amine chemical bonding. The antibody-fluorescence dye mixed solution was incubated for 1 h at room temperature in the dark, and desalting was performed with PD-10 desalting column to remove free dye. The fluorescent dye/protein (F/P) ratio was calculated using the NanoDrop spectrophotometer at 280 nm for antibodies and 495 nm for FITC, and the F/P ratio was 1.8 to 2.1.

### Cell lines

The HCT116 cell line was purchased from the American Type Culture Collection (ATCC; Manassas, VA, USA) and maintained in DMEM (HyClone, UT, USA) with 10% FBS (HyClone, UT, USA) and 1% Antibiotic–Antimycotic (AA; Gibco Laboratories, MD, USA). The FreeStyle 293-F cell line was purchased from Thermo Fisher Scientific (MA, USA). CCSP-2-overexpressing HCT116 cells (HCT116/CCSP-2) and control HCT116 (HCT116/pcDNA) cells were established from the transfection of CCSP-2-V5/His-tagged plasmid vectors with Lipofectamine 2000 (Invitrogen, CA, USA)^24^. Transfected cells were selected with 200 μg/mL geneticin (G418; Gibco Laboratories, MD, USA) in DMEM/F12 supplemented with 10% FBS and 1% AA.

### Immunoblot analysis

Colorectal cancer cell lysates were prepared in RIPA lysis buffer (Thermo Fisher Scientific, MA, USA) with protease inhibitor cocktail (Gendepot, Barker, TX, USA). Cell lysates and culture supernatant samples were separated by 10% SDS-PAGE and transferred to PVDF membranes. Protein-transferred membranes were blocked with 5% skim milk in Tris-buffered saline Tween 20 (TBS-T) blocking buffer for 1 h at room temperature and incubated at 4 °C overnight with blocking buffer-diluted anti-CCSP-2 IgG (1:1000) and anti-CCSP-2 scFv (1:1000) as primary antibodies. Anti-mouse IgG-HRP (1:2000; Cell Signaling Technology, MA, USA) for anti-CCSP-2 IgG and anti-human kappa IgG-HRP (1:2000; Novus Biologicals, Littleton, CO, USA) for scFv antibodies were used as secondary antibodies. Anti-β-actin antibody (Sigma-Aldrich Co., MO, USA) was used as the loading control. Protein expression signals were detected with ECL substrate (Thermo Fisher Scientific, MA, USA) and visualized using Luminograph III (ATTO Corporation, Tokyo, Japan).

### Xenograft anti-CCSP-2 scFv-FITC imaging

HCT116/CCSP-2 and HCT116/pcDNA cell suspensions in 100 μL of PBS with Matrigel (Corning, NY, USA) were subcutaneously injected into 6- to 8-week-old balb/c nude mice (Orient Bio, Seongnam, Korea). After 20 days, the mice were examined by molecular imaging. The CCSP-2-targeted (n = 6) or control (n = 4) probe was intravenously injected into the tail vein of xenograft mice (1.5 μg/g body weight), and fluorescence images were acquired at 2, 4, 6, and 8 h after i.v. injection. For biodistribution, the molecular images of isolated organs (heart, liver, kidney, spleen, lung, and colon) were acquired immediately after the mice were sacrificed. The molecular imaging of xenograft tumors was performed using the Xenogen IVIS Spectrum system (Caliper Life Sciences, MA, USA).

### Immunofluorescence localization of anti-CCSP-2 scFv in tissues and cells

Immunofluorescence staining of CCSP-2 was performed by seeding HCT116/CCSP-2 and HCT116/pcDNA cells (30,000 cells/well) in 8-chamber slides (Thermo Fisher Scientific, MA, USA) and fixation with 4% paraformaldehyde. The cells were washed with PBS and blocked with 5% normal goat serum (Cell Signaling Technology, MA, USA) in 1% BSA in PBS-Tween 20 (PBS-T) as the dilution buffer for 60 min. FITC-conjugated anti-CCSP-2 scFv and control scFv (5 μg/mL) in dilution buffer were added to each cell line for 60 min after washing with PBS. PBS-washed cells were incubated with 4,6-diamidino-2-phenylindole (DAPI; Invitrogen, CA, USA) to stain the nucleus for 10 min, and the cells were visualized with a LSM880 confocal microscope (Carl Zeiss, Jena, Germany).

### Molecular colonoscopic imaging of orthotopic colon cancer mice

Before orthotopic injection was performed, a combined needle was prepared for insertion through the working channel of the colonoscope (Vetcom; Karl Storz, Tuttlingen, Germany). A 30G needle was bound to a 23G needle with a flexible plastic pipe. Then, 1 × 10^7^ HCT116/CCSP-2 cells in 50–100 µL of 10% Matrigel (Corning)/PBS were injected into 6- to 8-week-old balb/c mice (Orient Bio, Seongnam, Korea). Colonic submucosal injection was carefully performed through the working channel of the endoscope. After injection, colonoscopic imaging was performed every week to confirm tumor formation (Video S1A). Colonoscopy was performed using the IMAGE1 H3-Z F1 THREE-CHIP FULL HD Camera System (Part TH102), Image 1 HUB CCU (Parts TC200EN, TC300), Modified D-Light P VET Source (Part 66100M3), AIDA HD Control System, STRAIGHT FORWARD (Part 64301AA), and fluorescent filters (Karl Storz, Tuttlingen, Germany). All acquired optical and fluorescence colonoscopy images were recorded as MP4 video files by the AIDA HD control system (Karl Storz, Tuttlingen, Germany). For i.v. administration, the FITC-conjugated anti-CCSP-2 scFv probe was intravenously injected into the tail vein of orthotopic mice (1.5 μg/g body weight). Molecular (FITC) imaging was performed at 0, 2, 4, and 6 h after injection by fluorescence colonoscopy (Vetcom; Karl Storz, Tuttlingen, Germany). In addition, the molecular images of isolated organs (colon, heart, liver, kidney, spleen, and lung) were acquired using the Xenogen IVIS Spectrum System (Caliper Life Sciences, MA, USA) immediately after the mice were sacrificed at 6 h after injection. For topical administration, the FITC-conjugated anti-CCSP-2 scFv probe (30 μg/mL) was intrarectally administered to the orthotopic mice. Fluorescence colonoscopy (Vetcom; Karl Storz, Tuttlingen, Germany) was performed at 10–15 min after administration.

### Single-dose toxicity and total fluorescence half-life analysis

FITC-conjugated scFv (1.5 µg/g body weight) was intravenously injected into the tail vein of 18 healthy balb/c nude mice (Orient Bio, Seongnam, Korea), and blood samples were collected from the abdominal vein. The blood samples were used for hematologic analysis, and some of the blood samples were separated into the plasma fraction and used for chemical analysis (Supplementary Table S2).

The half-life of intravenously injected anti-CCSP-2 scFv-FITC was measured at 0, 2, 4, 6, and 8 h after injection using mouse whole-body fluorescence images^44^.

### Ex vivo molecular imaging of human colorectal tumors

Adenocarcinoma and adjacent normal tissues excised from colorectal cancer patients were washed with ice-cold PBS. Excised tissues were loaded using low-melting agarose gels at room temperature until solidified to prevent false positive detection from the dissected tissue surface by sprayed FITC-conjugated scFv antibodies. Incubation with FITC-conjugated scFv antibodies (30 μg/mL) diluted in 1% BSA in PBS-T was carried out at room temperature for 30 min. Tissues loaded on agarose were washed with PBS-T and detected with the Xenogen IVIS Spectrum System (Caliper Life Sciences, MA, USA) at an excitation wavelength of 500 nm and an emission wavelength of 540 nm.

### Immunohistochemistry of anti-CCSP-2 IgG in tissues

Immunohistochemistry was performed as described previously^24^. In brief, patient colon tissues were fixed with 4% paraformaldehyde and embedded in paraffin. The BenchMark XT automatic immunostaining device (Ventana Medical Systems, AR, USA) and OptiView DAB IHC Detection Kit (Ventana Medical Systems, AR, USA) were used for immunostaining. Tissue sections (4 μm) were transferred to salinized, charged slides and incubated at room temperature and 65 °C. After epitope retrieval for 64 min, the sections were incubated in an automatic immunostainer with anti-CCSP-2 IgG (1:100) for 32 min. The staining patterns of the slides were visualized with OptiView DAB IHC Detection Kit (Ventana Medical Systems, AR, USA).

### Statistical methods

Data are presented as the mean ± standard error of the mean (SEM), which were analyzed by paired t-test to determine significant differences with GraphPad Prism (GraphPad Software, CA, USA).

## Supplementary Information

Supplementary Information 1.

Supplementary Video 1.

Supplementary Video 2.

Supplementary Video 3.
