# Supplementary material for: Biomolecular imaging of colorectal tumor lesions using a FITC-labeled scFv-Cκ fragment antibody
Source: Sci Rep. 2021 Aug 25;11:17155. doi: 10.1038/s41598-021-96281-z (PMC8387423; doi:10.1038/s41598-021-96281-z)
Supplement: Supplementary file 1 — Supplementary Information 1. [file 41598_2021_96281_MOESM1_ESM.pdf]

Biomolecular imaging of colorectal tumor lesions using a FITC labeled scFv-Cκ fragment antibody

Hyung il Kim[a,g*], Jinhyeon Kim[b*], Hyori Kim[c], Hyeri Lee[b], Yong Sik Yoon[d], Sung Wook Hwang[e], Sang Hyoung Park[e], Dong-Hoon Yang[e], Byong Duk Ye[e], Jeong-Sik Byeon[e], Suk-Kyun Yang[e], Sun Young Kim[f†], Seung-Jae Myung[b],[e,f,g†]

[a]*Department of Medical Science, Asan Medical Institute of Convergence Science and Technology, Asan Medical Center, University of Ulsan College of Medicine, Seoul, Korea*

[b]*Edisbiotech, Songpa-gu, Seoul, Korea*

[c]*Convergence Medicine Research Center, Asan Medical Center, Seoul, Republic of Korea*

[d]*Department of Colon and Rectal Surgery, Asan Medical Center, University of Ulsan College of Medicine, Seoul, Korea*

[e]*Department of Gastroenterology, Asan Medical Center, University of Ulsan College of Medicine, Seoul, Korea*

[f]*Asan Institute for Life Sciences, Asan Medical Center, University of Ulsan College of Medicine, Seoul, Koreag*

[g]*Digestive Diseases Research Center, University of Ulsan College of Medicine, Seoul, Korea*

†Corresponding Author:

88, Olympic-ro 43-gil, Songpa-gu, Seoul 05505, Korea

E-mail: enthalpy98@gmail.com; sjmyung@amc.seoul.kr

*contributed equally to this work.

Supplementary Figure S1.

A

LTQPSSVSANPGETVEITCSGGSNSYYGWYQQKSPGSAPVTVIYDNTNRPSNIPSRFSGSTSGST
STLTITGVQADDEAVYYCGSTDSSYVDIFGAGTTLTVL

B

AVTLDESGGGLQTPGGALSLVCKASGFSFSGVNMHWVRQAPGKGLEYVAQISSTGSGTGYGSAVQG
RATISRDNGQSTVRLQLNNLRAEDTGIYYCAKDAYGYRISGSWSYGYSIDAWGHGTEVIVSSTS

Supplementary Figure S1. Amino acid sequences of anti-CCSP-2 antibody variable region. The amino acid sequence of the light chain variable region (A) and heavy chain variable region (B) are shown. Red letters indicate complementary determining regions (CDR) of each variable region (25.64kDa, *pI*=pH 4.87).

Supplementary Figure S2.

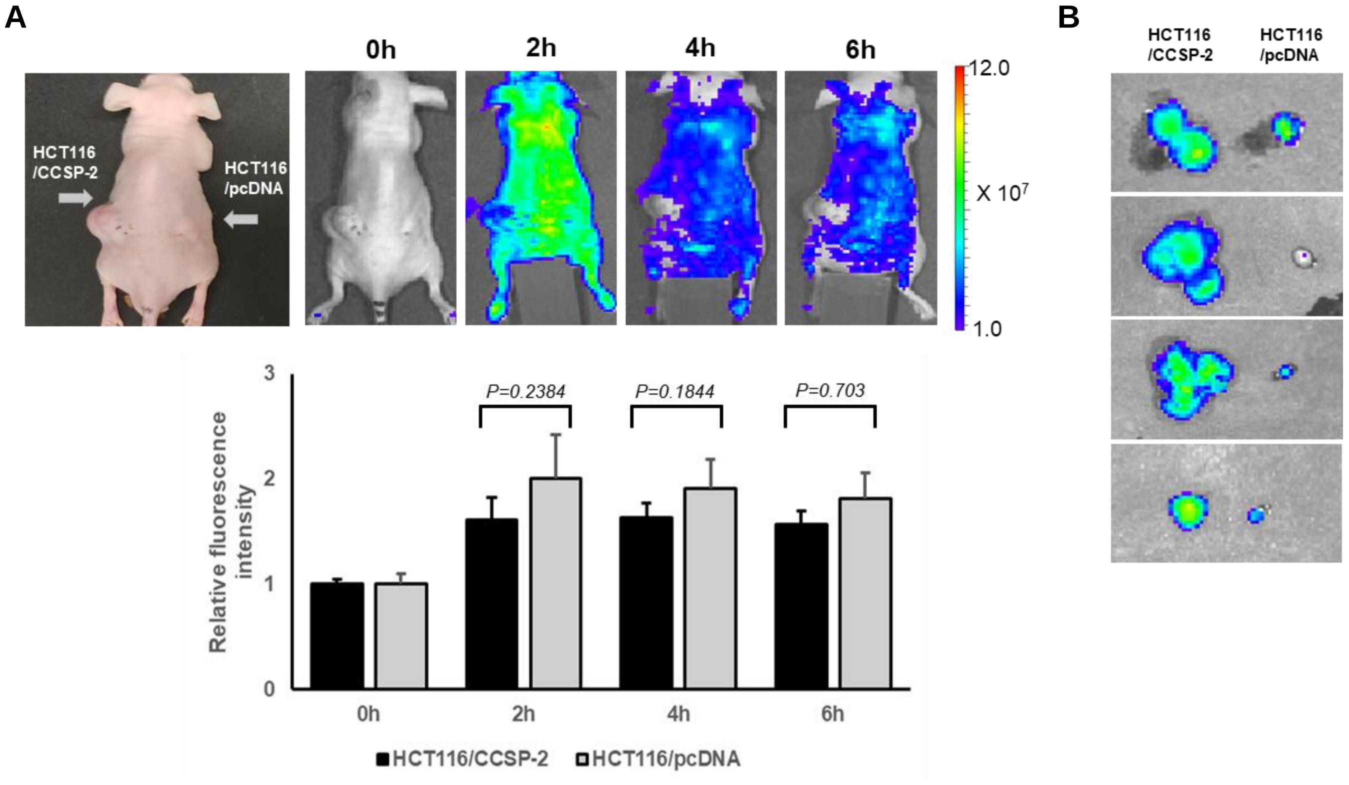

Supplementary Figure S2. (A) In vivo molecular images of CCSP-2-overexpressing xenograft tumor-bearing mice after the tail vein injection of control scFv-FITC (B) Isolated solid tumors were acquired from sacrificed mice to examine the fluorescence intensity ex vivo.

Supplementary Figure S3.

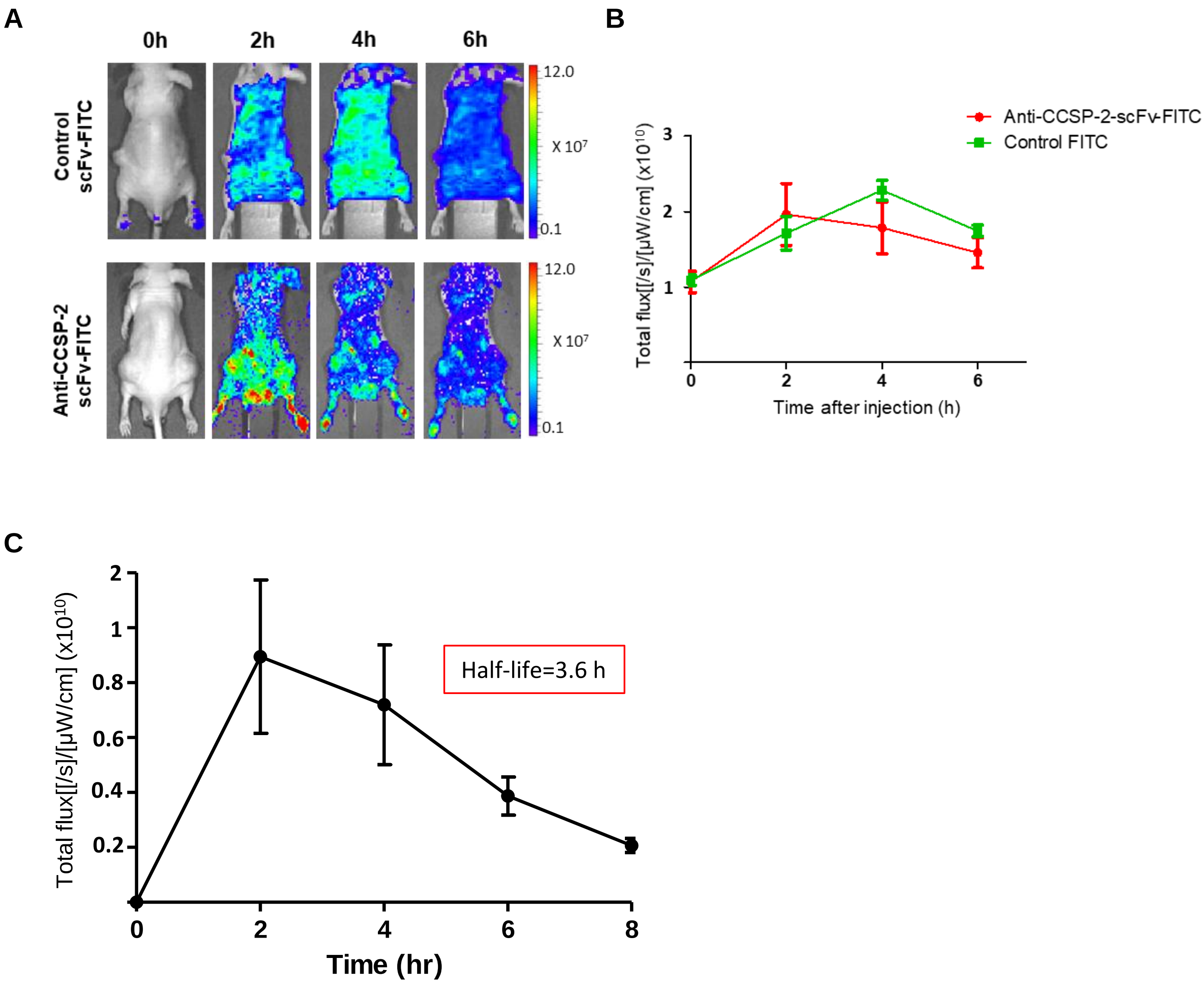

Supplementary Figure S3. Total fluorescence intensity after the tail vein injection of anti-CCSP-2 scFv-FITC or control scFv-FITC.

Supplementary Figure S4.

A

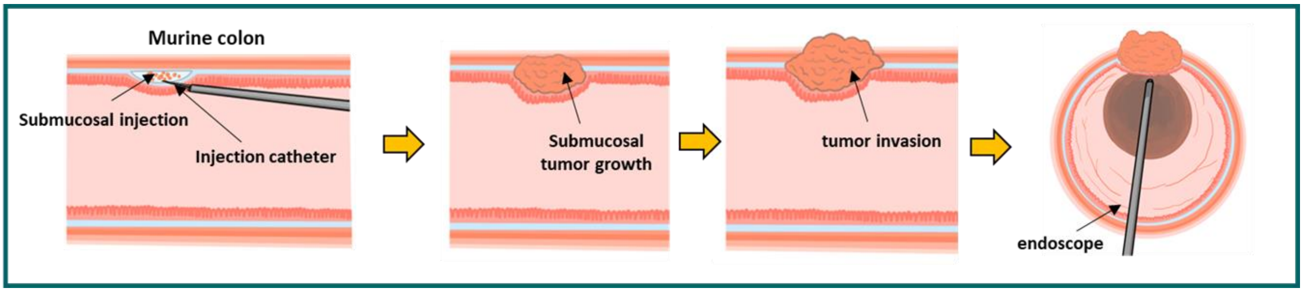

B

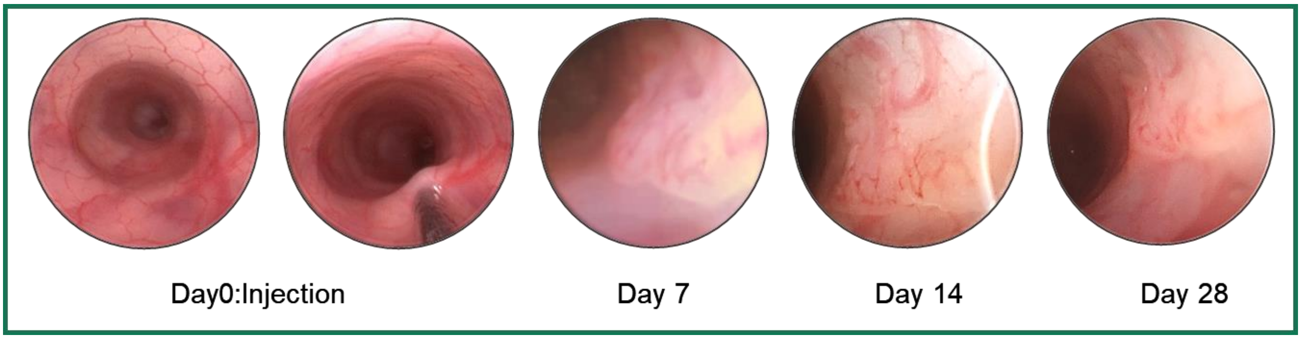

Supplementary Figure S4. Establishment of an orthotopic murine colorectal cancer model. (A),(B) Implanted tumor growth was monitored by regular endoscopic examination over a follow-up period of 28 days. CCSP-2-overexpressing cells (1×10^{7}) in 10% Matrigel/PBS were used for colonic submucosal injection, and the tumors were examined by white-light colonoscopy.

Supplementary Figure S5.

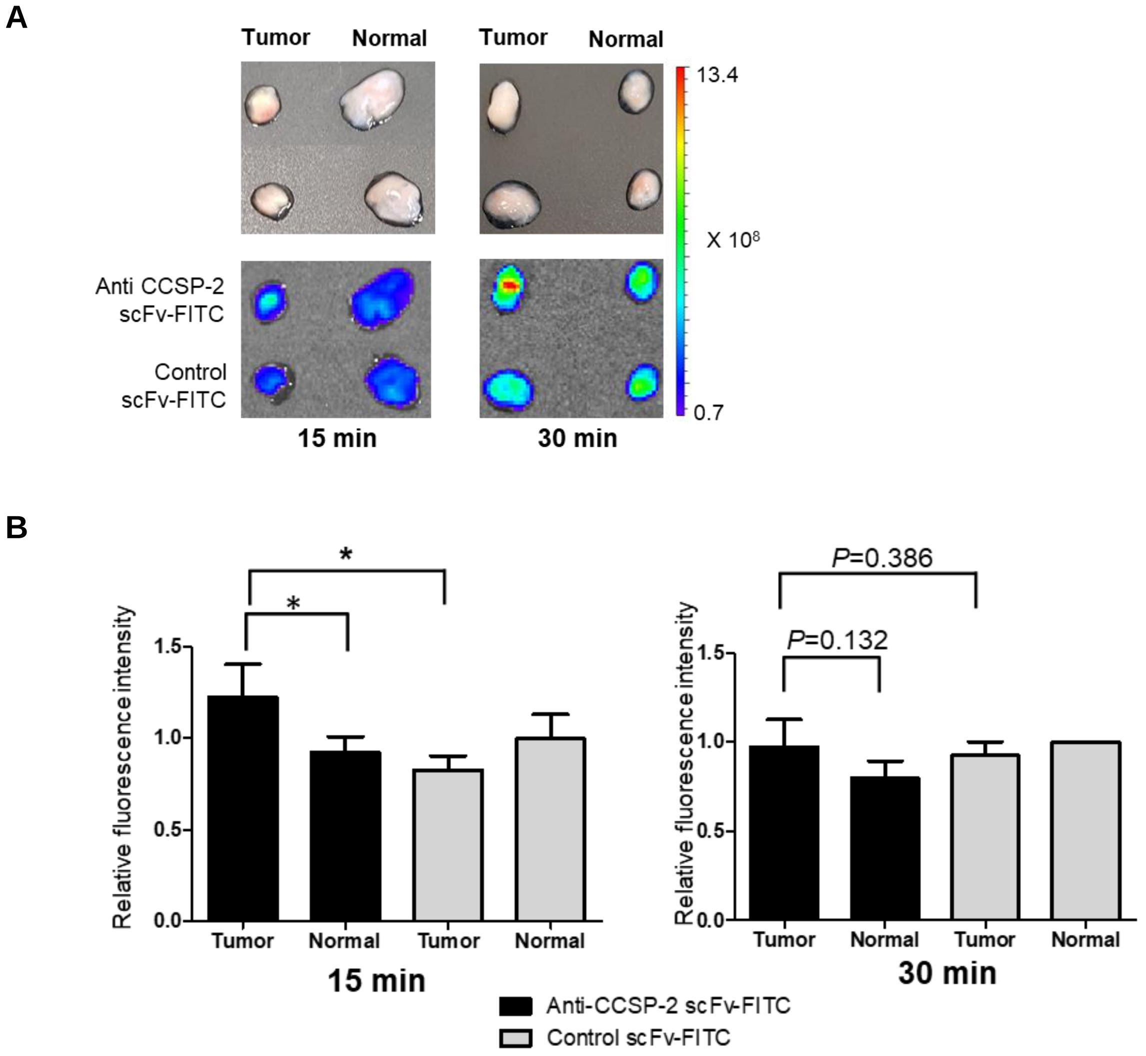

Supplementary Figure S5. Ex vivo fluorescence imaging in human frozen colorectal cancer specimens with anti-CCSP2-scFv (A) Representative images of of anti-CCSP-2 scFv-FITC or control scFv-FITC treated frozen colorectal cancer specimens. (B) Relative fluorescence intensity graph of colorectal cancer patient specimens treated with FITC-conjugated anti-CCSP-2 scFv and control scFv (n =5 , *p < 0.05).

Supplementary Figure S6.

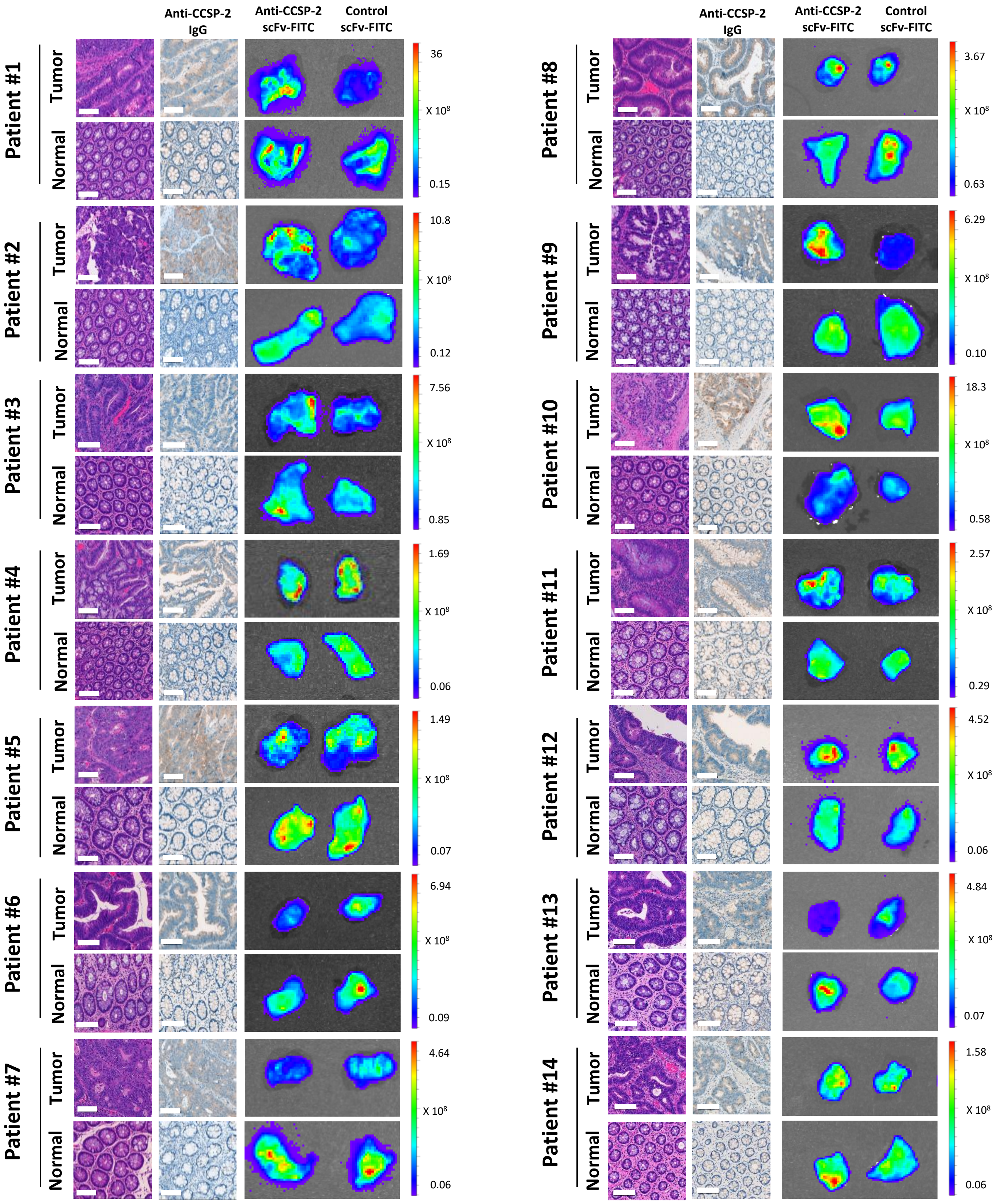

Supplementary Figure S6. Histological analysis of colorectal tumor and normal tissues from colorectal cancer patients with H&E, IHC and *ex vivo* immunofluorescence imaging (original magnification, 100×, scale bar: 100 μm, fluorescence intensity unit: [p/s/cm^2/sr]/[μW/cm^2], n=14).

Supplementary Figure S7.

A

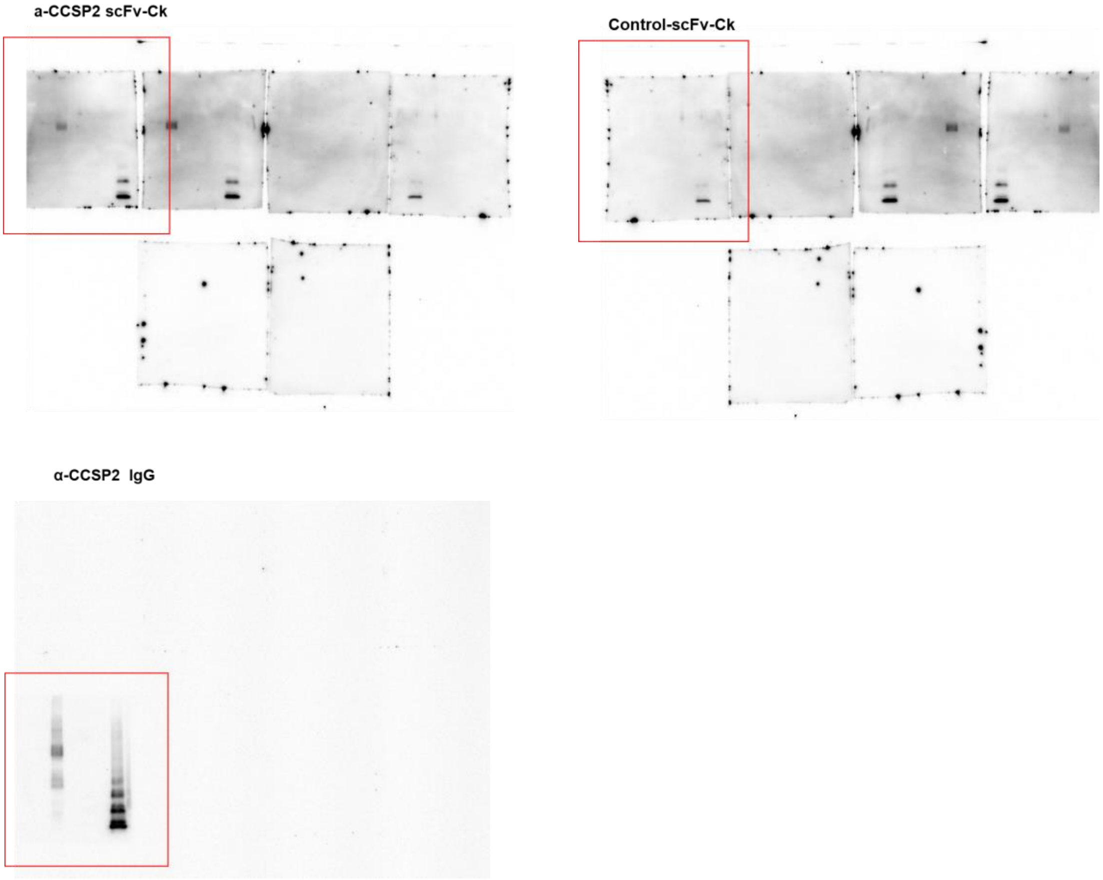

B

Culture sup. (secreted)

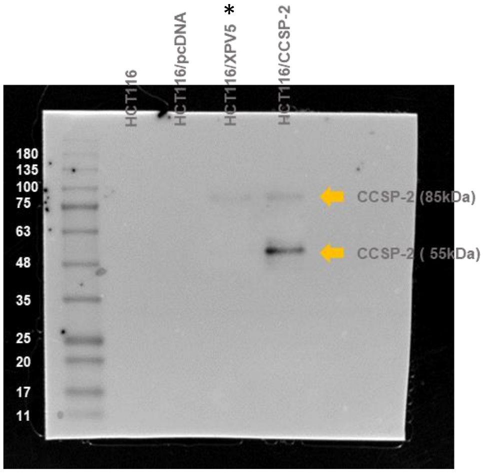

Cell lysate

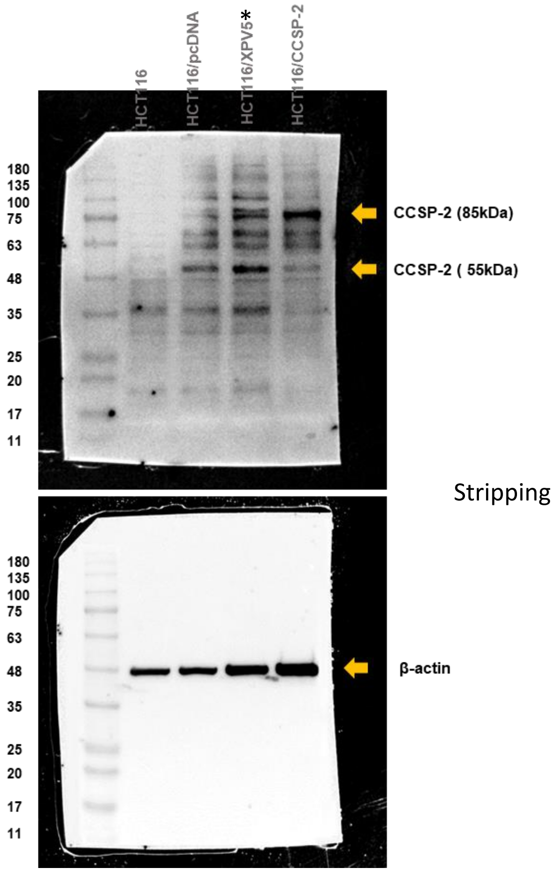

* HCT116/XPV5 is bulk amplified stable cell line and HCT116/CCSP-2 is from single colony amplification. We used only HCT116/CCSP-2 for experiment so that HCT116/XPV5 was cropped in main figure.

Supplementary Figure S7. Uncropped western blot images used in Figure 1 (A) and Figure 2 (B).

Supplementary Table S1.

Antibody	ELISA K_D(nM)	EC_{50}(nM)	EC_{50}(ng/ml)	Std. Error
α-CCSP2 IgG	3.2	3.5	186.8	0.023
α-CCSP2 scFv-Cκ	5.2	5.0	260.9	0.016

Supplementary Table S2. Clinical signs, hematology, and clinical chemistry data for mice in the single dose toxicity study

	**Normal Range**	Control	6 hrs	24 hrs
n		8	5	5
**Male**				
No observable abnormality		8	5	5
Death		0	0	0
White blood cells (10^3/μL)	(3.48 - 14.03)	*6.3 ± 2.30*	*4.1 ± 1.40*	*5.2 ± 2.95*
Red blood cells (10^6/μL)	(6.93 - 12.24)	*9.2 ± 0.67*	*8.9 ± 0.59*	*9.2 ± 0.54*
Hemoglobin (g/dL)	(12.6 - 20.5)	*13.4 ± 0.90*	*13.3 ± 0.75*	*13.4 ± 0.59*
Hematocrit (%)	(42.1 - 68.3)	*43.6 ± 3.12*	*43.5 ± 3.08*	*44.9 ± 2.04*
Mean corpuscular volume (fL)	(42.2 - 59.2)	*47.2 ± 0.96*	*48.9 ± 1.11*	*48.9 ± 0.76*
Mean corpuscular hemoglobin (pg)	(13.2 - 17.6)	*14.6 ± 0.44*	*15.0 ± 0.19*	*14.7 ± 0.24*
Mean corpuscular hemoglobin concentration (g/dL)	(23.3 - 32.7)	*28.7 ± 5.93*	*30.7 ± 0.86*	*30.1 ± 0.32*
Red cell distribution width (%)	(11.7 - 15.1)	*12.6 ± 4.56*	*13.7 ± 0.44*	*13.6 ± 0.37*
Hemoglobin distribution width (g/dL)	(1.8 - 2.6)	*2.2 ± 0.22*	*2.0 ± 0.22*	*2.0 ± 0.07*
Platelet (10^3/μL)	(420 - 1698)	*1078.6 ± 362.23*	*733.2 ± 239.06*	*1042.8 ± 162.13*
Mean platelet volume (fL)	(5.0 - 8.0)	*10.4 ± 1.34*	*7.0 ± 2.45*	*9.3 ± 1.00*
% Neutrophils	(9.8 - 39.1)	*39.5 ± 14.48*	*43.2 ± 5.24*	*39.2 ± 8.91*
% Lymphocytes	(48.8 - 83.2)	*53.6 ± 15.69*	*47.4 ± 5.55*	*53.1 ± 8.35*
% Monocytes	(3.29 - 12.48)	*3.4 ± 0.82*	*3.3 ± 0.94*	*4.6 ± 2.02*
% Eosinophils	(0.1 - 4.91)	*2.8 ± 1.55*	*5.4 ± 1.67*	*2.4 ± 1.02*
% Basophils	(0 - 1.58)	*0.1 ± 0.08*	*0.3 ± 0.21*	*0.1 ± 0.04*
% Large unstained cells	(0 - 3.2)	*0.6 ± 0.22*	*0.5 ± 0.17*	*0.6 ± 0.37*
Aspartate Aminotransferase (U/L)	(55 - 352)	*68.5 ± 35.57*	*70.0 ± 5.45*	*56.5 ± 18.60*
Creatinine (mg/dL)	(0.2 - 0.4)	*0.3 ± 0.03*	*0.3 ± 0.03*	*0.3 ± 0.02*

Supplementary Video S1. Orthotopic-mucosal injection by using working channel of the colonoscope

Orthotopic-mucosal injection

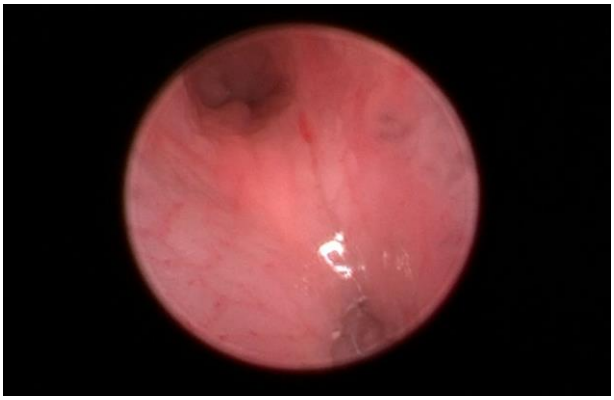

Supplementary Video S2. Fluorescence colonoscopic imaging of anti CCSP-2 scFv-FITC in colorectal cancer from orthotopic mouse model

Orthotopic-CCSP-2-scFv-FITC

A

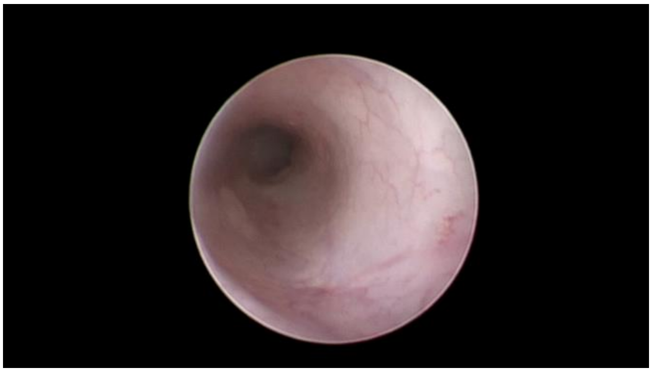

Bright field colonoscopy

B

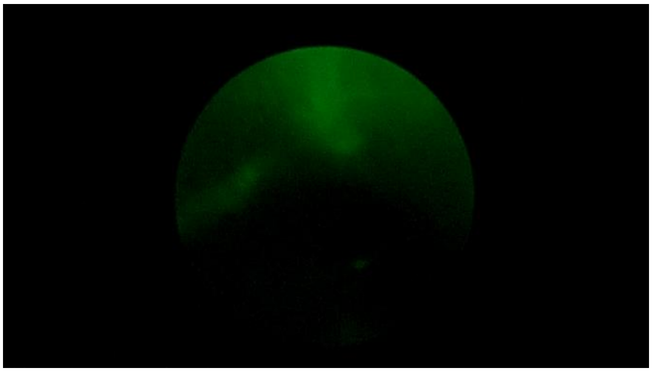

Fluorescence colonoscopy
